# Surgeon Estimations of Acetabular Cup Orientation Using Intraoperative Fluoroscopic Imagining Are Unreliable

**DOI:** 10.1016/j.artd.2023.101109

**Published:** 2023-03-07

**Authors:** Parker L. Brush, Adrian Santana, Gregory R. Toci, Eric Slotkin, Michael Solomon, Tristan Jones, Arjun Saxena

**Affiliations:** a Rothman Orthopaedic Institute at Thomas Jefferson University, Philadelphia, PA, USA; b Rutgers Robert Wood Johnson Medical School, New Brunswick, NJ, USA; c Orthopaedic Associates of Reading, Tower Health, Reading Hopsital, West Reading, PA, USA; d Sydney Orthopaedic Specialists, Prince of Wales Private Hospital, Randwick, Australia; e Corin Group, Cirencester, Gloucestershire, UK

**Keywords:** Total hip arthroplasty, direct anterior approach, intraoperative fluoroscopy, acetabular anteversion, acetabular inclination

## Abstract

**Background:**

Accurate acetabular cup orientation is associated with decreased revision rates and improved outcomes of primary total hip arthroplasty. This study assesses surgeon’s ability to estimate both the acetabular component inclination and anteversion angles via intraoperative fluoroscopy (IF) images.

**Methods:**

We surveyed orthopedic surgeons to estimate acetabular component inclination and anteversion based on 20 IF images of total hip arthroplasty through a direct anterior approach. Postoperative computed-tomography scans were used to calculate the true inclination and anteversion component angles. The absolute difference between the true and estimated values was calculated to determine the mean and standard deviation of the survey results. Interrater reliability was determined through interclass correlation coefficients.

**Results:**

A majority of surgeons preferred the direct anterior approach (83.3%) and utilized IF during surgery (70%). Surgeons surveyed were on average 5.9° away from the true value of inclination (standard deviation = 4.7) and 8.8° away from the true value of anteversion (standard deviation = 6.0). Respondents were within 5° of both inclination and anteversion in 19.7% of cases, and within 10° in 57.3% of cases. All surgeons were determined to have poor reliability in estimating anteversion (interclass correlation coefficient < 0.5). Only 2 surgeons were determined to have moderate reliability when estimating inclination.

**Conclusions:**

Surgeons, when solely relying on IF for the estimation of anteversion and inclination, are unreliable. Utilization of other techniques in conjunction with IF would improve observer reliability.

## Introduction

Total hip arthroplasty (THA) is one of the most commonly performed orthopedic procedures with an estimated 572,000 primary THAs expected to be performed in 2030 [1,2]. Surgeons are performing more THAs through the direct anterior approach (DAA), with more than 50% of surgeons utilizing the approach [3,4]. One of the most important outcome measures in arthroplasty surgery is revision rate [5]. Authors have reported revisions rates for THA from <5% to 12.9% at 10 years, with aseptic loosening and dislocation as the most common reasons for revision [[5], [6], [7], [8], [9], [10]]. However, meta-analyses report lower rates of dislocation with the DAA [11,12]. Other studies have identified associations between improper component positioning and resultant increases in leg-length discrepancy, impingement, imbalanced stress distribution, and dislocations [[13], [14], [15], [16]]. In order to improve outcomes, researchers have suggested that the use of intraoperative fluoroscopy (IF) for the DAA has helped to improve acetabular cup orientation; however, surgeons must take measures to limit the impact of pelvic tilt on intraoperative measurements [17,18]. Regardless, surgeons commonly use IF to assist in bone preparation, component position, and intraoperative leg-length measurements [19]. Given the importance of component positioning in THA and the reliance on IF, we set out to answer if (1) surgeons are reliable in assessing acetabular orientation based on IF and (2) how often are surgeon estimations off by 5°, 10°, and 20°. Our hypothesis is that visual inspection of IF is inadequate in estimating acetabular component positioning.

## Material and methods

**Figure 1:**
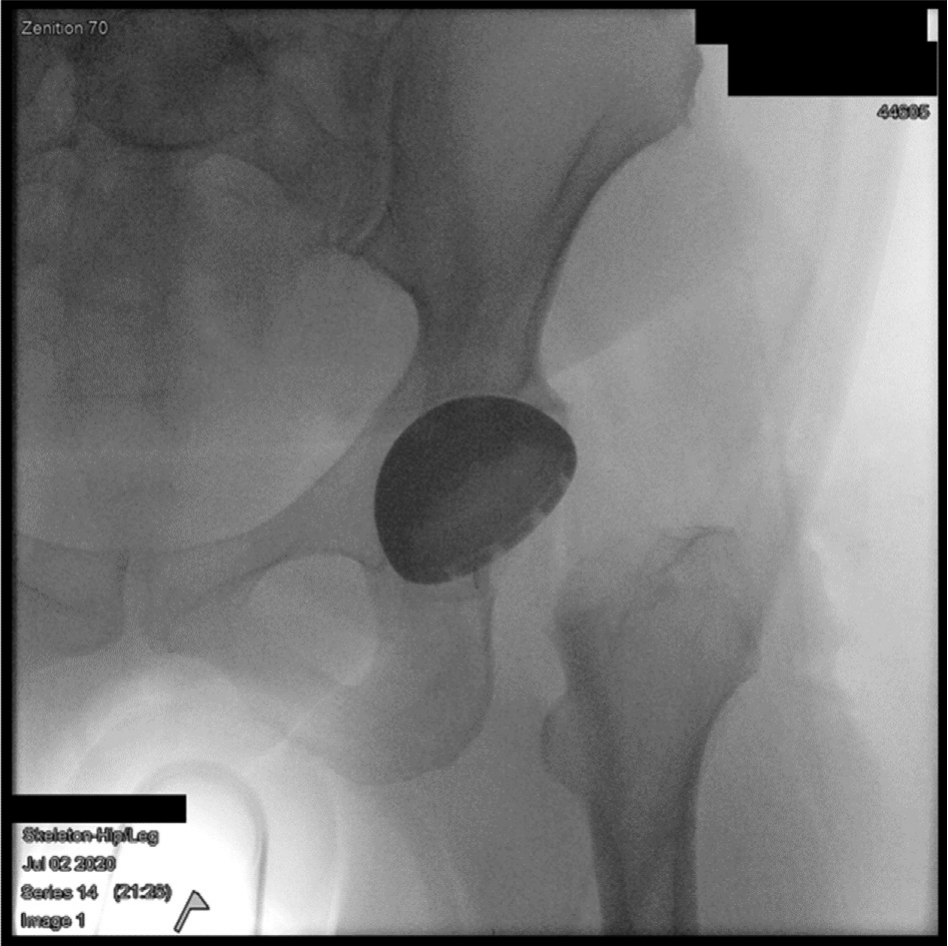
Example intraoperative fluoroscopic image used in the survey.

**Figure 2:**
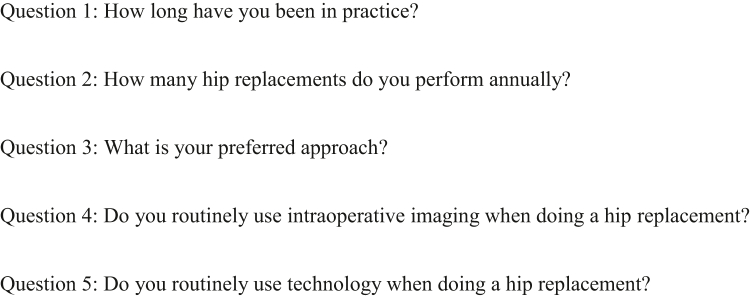
List of the general questions included in the survey.

**Supplemental Figure 1:**
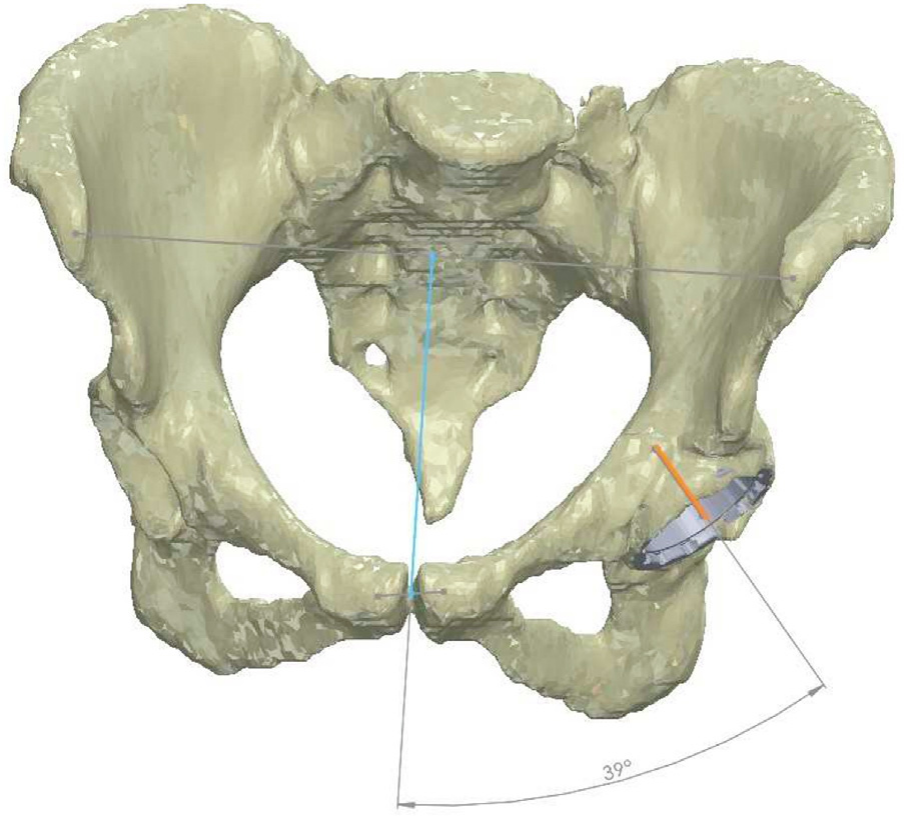
Example image of how the 3D reconstruction and measuring software were able to determine acetabular cup inclination.

**Supplemental Figure 2:**
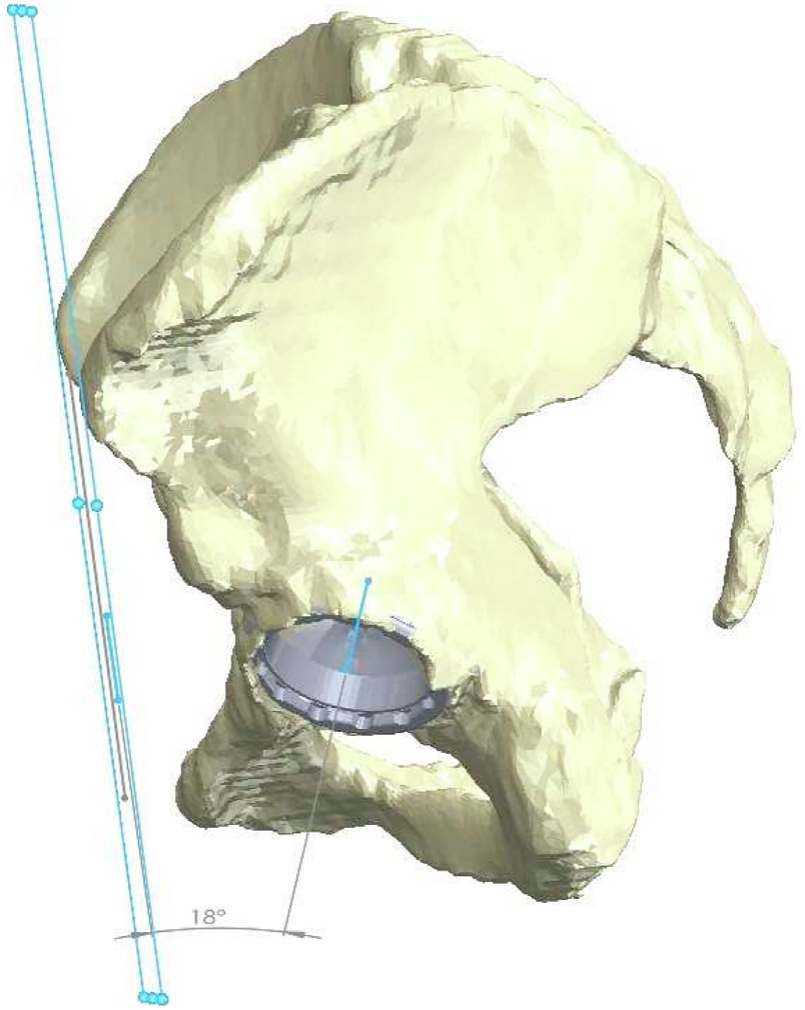
Example image of how the 3D reconstruction and measuring software were able to determine acetabular cup anteversion.

We retrospectively identified 30 patients who underwent primary THA by a DAA from 2 surgeons with both IF and a postoperative computed-tomography (CT) scan on record. Patients who had incomplete image data, received a revision THA, or a THA through an approach other than direct anterior were excluded. We used the Simpleware ScanIP (Synopsis, Mountain View, CA) software to extract anatomical landmarks from postoperative CT scans and the Solidworks (Dassault Systèmes, France) software to compute values for the true inclination and anteversion of the acetabular component. The Corin Group (Cirencester, UK) constructed a survey in coordination with the authors of the manuscript consisting of 5 general questions and 20 blinded, anterior-posterior IF images in which the participants were requested to estimate the acetabular component inclination and anteversion. This estimation was performed by visual inspection of the fluoroscopic images in order to simulate the operative environment, no assist tools were allowed to be used, and no practice images were provided. Figure 1 contains an example of one of the IF images used in the survey, and Supplemental Figures 1 and 2 contain the corresponding inclination and anteversion measurements performed on the 3D pelvis reconstruction, respectively. We removed 10 patients from the survey at random to increase the response rate and ease the time burden required to complete the survey. Figure 2 contains the 5 general questions included in the survey, and Table 1 includes the categorical options provided as answers. We sent the survey on 1 occasion to 89 surgeons in 3 different hospital systems with a combination of fellowship-trained arthroplasty surgeons, arthroplasty fellows in training, and postgraduate-year-four and postgraduate-year-five residents. The survey was sent broadly to all the senior authors’ contacts to maximize the potential responses. The surgeons’ responses were collected between July 2021 and January 2022.

**Table 1:** Responses to survey questions 1 through 5.

Full cohort	N = 30
Length of practice	
<5 y	11 (36.7%)
5-15 y	13 (43.3%)
15-25 y	4 (13.3%)
>25 y	2 (6.67%)
THA per year	
<50	3 (10.0%)
50-100	4 (13.3%)
100-200	14 (46.7%)
>200	9 (30.0%)
Preferred approach	
Direct anterior	25 (83.3%)^a^
Posterolateral	6 (20%)^a^
Anterolateral	1 (3.3%)
Routinely use intraoperative imaging	
No	9 (30.0%)
Yes	21 (70.0%)
Routinely use technology	
Digital preoperative planning	21 (70%)
Patient-specific instrumentation	2 (6.7%)
Imageless navigation	3 (10%)
Image-based navigation	4 (13.3%)
Intraoperative imaging with digital measurements	1 (3.3%)
Other	3 (10%)

THA, total hip arthroplasty.

a Some responses included multiple answers.

We calculated absolute differences for each inclination and anteversion response by taking the absolute value of the difference between the true value determined by postoperative CT scans and the responses in the survey. The responses were analyzed by 2 methods: (1) The surgeon’s responses to each image were considered separately, and (2) the surgeon’s responses to the image set were averaged together. To account for nonresponse bias, an additional analysis between early and late responders was performed under the assumption that late responders are most similar to nonresponders [20].

An independent statistician performed the statistical analysis using R Studio (Version 4.1.2, Vienna, Austria). They analyzed the survey data by intraclass correlation coefficients (ICC) to evaluate for interrater reliability of responses compared to true values. ICC values less than 0.5 indicate weak agreement, values from 0.5 to less than 0.7 indicate moderate agreement, and values greater than or equal to 0.7 indicate strong agreement with the true values. Each survey respondent has an ICC value associated with their inclination and anteversion responses.

## Results

We received 34 responses to the survey for a response rate of approximately 38%. Four responses were removed as they were incomplete. All complete responses were included in the final analysis.

Table 1 contains the categorical responses to survey questions 1 through 5. We found most participants preferred the DAA (83.3%), routinely used IF (70%), and routinely used a preoperative planning system (66.7%). Two of the participants reported that they prefer the direct anterior and posterolateral approaches equally. Three survey participants wrote in answers for “other” use of technology. Their responses were (2) optimized positioning system and (1) digital preoperative planning combined with conventional instruments.

Table 2, Table 3 contain descriptive statistics for the cumulative inclination and anteversion survey values with an analysis performed at both the individual response and surgeon average level. We based all descriptive statistics on the absolute differences. On average, surgeons were 5.9° away from the true value for inclination and 8.8° away from the true value for anteversion with standard deviations of 4.7° and 6.0° at the individual response level and 1.7° and 2.4° at the surgeon average level, respectively. Our survey responses ranged from 0 to 39 for inclination and 0 to 29 for anteversion by absolute difference. We identified 118 (19.7%) responses within 5° of both the true anteversion and inclination, and 344 (57.3%) of responses within 10°. Out of the 600 data points, 28 (4.7%) surgeons were off by 20° or greater for anteversion, and 8 (1.3%) were off by 20° or greater for inclination, with a total of 32 (5.3%) patients with either an anteversion or inclination estimation off by 20° or more. This value was chosen based on the historical safe zone of component position [21].

**Table 2:** Descriptive statistics for the component inclination by absolute value.

Individual response inclination		
Range	0-28	
Mean	5.9	
Median	5.0	
SD	4.7	
Surgeon average inclination		
Range	3.3-9.1	
Mean	5.9	
Median	5.7	
SD	1.7	

SD, standard deviation.

**Table 3:** Descriptive statistics for the component anteversion by absolute value.

Individual response anteversion	
Range	0-39
Mean	8.8
Median	9.0
SD	6.0
Surgeon average anteversion	
Range	6.0-15.6
Mean	8.8
Median	8.0
SD	2.4

SD, standard deviation.

Table 4 contains the ICC values for each participant with regard to inclination and anteversion estimations. We removed participants 15, 16, 26, and 27 for incomplete responses. All surgeons had a weak agreement with the true values for component anteversion. Two surgeons had a moderate agreement with the true values with regard to component inclination, participants 18 and 22. These participants were in practice between 5 to 15 and less than 5 years, performed 100 to 200 and less than 50 THAs per year, respectively, routinely used intraoperative imaging, and used digital preoperative planning systems. The remainder of surgeons had a weak agreement with regard to component inclination. We found no surgeons to have strong agreement by ICC values. Given the consistently low reliability among survey participants, we determined that increasing our sample size would not likely impact the results of this study, and the survey was closed.

**Table 4:** ICC values for each participant.

Survey participant	Inclination	Anteversion
1	−0.06	−0.04
2	0.38	−0.05
3	0.34	0.02
4	0.28	0.09
5	0.26	0.10
6	0.00	0.22
7	0.14	0.19
8	0.25	−0.02
9	0.38	0.07
10	0.27	0.18
11	0.21	−0.02
12	−0.07	0.05
13	0.13	0.06
14	0.01	0.22
17	0.28	0.03
18	0.67^a^	0.17
19	−0.12	0.11
20	0.01	0.11
21	0.19	0.30
22	0.53^a^	−0.20
23	0.13	0.09
24	0.16	0.16
25	0.27	−0.31
28	0.31	0.11
29	0.10	0.07
30	−0.11	0.08
31	0.16	−0.10
32	0.36	0.04
33	0.29	0.02
34	0.19	0.17

ICC, interclass correlation coefficient.

a Moderate agreement with the true values.

Table 5 contains the analysis of the initial 5 (16.7%) and final 5 (16.7%) participants. These data show similar responses between the initial and final 5 participants by absolute inclination (6.3 vs 5.5, *P* = .511) and absolute anteversion (9.6 vs 8.7, *P* = .581).

**Table 5:** Comparisons of initial and final participants.

Characteristics	Initial N = 5	Final N = 5	*P* value
Length of practice			.048
<5 y	0 (0.00%)	2 (40.0%)	
5-15 y	5 (100%)	1 (20.0%)	
15-25 y	0 (0.00%)	1 (20.0%)	
>25 y	0 (0.00%)	1 (20.0%)	
THA per year			1.000
50-100	0 (0.00%)	1 (20.0%)	
100-200	3 (60.0%)	2 (40.0%)	
>200	2 (40.0%)	2 (40.0%)	
Intraoperative imaging			.444
No	0 (0.00%)	2 (40.0%)	
Yes	5 (100%)	3 (60.0%)	
Surgical technology			1.000
No	1 (20.0%)	2 (40.0%)	
Yes	4 (80.0%)	3 (60.0%)	
Average inclination	6.3 (1.69)	5.5 (2.06)	.511
Average anteversion	9.6 (2.25)	8.7 (2.66)	.581

Average inclination and average anteversion are provided as the average absolute difference from the true measurement.

THA, total hip arthroplasty.

## Discussion

This survey includes the responses of 34 surgeons that were asked to evaluate the acetabular cup orientation of 20 patients who underwent THA by DAA. We found most surgeons utilized and preferred the DAA with IF in our population. Moreover, approximately two-thirds of the surveyed surgeons routinely used digital preoperative planning systems, which has been shown to decrease time under fluoroscopy, especially when pelvic tilt is calculated [12].

Our primary finding is that all surgeons had poor agreement with the true values of component anteversion, while all but 2 surgeons had poor agreement with the true values of component inclination determined by the analysis of postoperative CT scans. These data indicate that the surgeons surveyed were not reliable in their determination of both component inclination and anteversion through IF images. This finding is supported by the previous work of Holst et al. who found that IF did not improve acetabular cup positioning or sizing when employing the DAA and differs from the work by James et al. who suggest that IF can help confirm component positioning if used properly [12,18]. Although no statistical measure interprets a correlation between the baseline characteristics we obtained and ICC values, we were unable to identify any associations between improved component orientation estimation and surgeon length of practice or total surgeries performed per year as almost every surgeon had statistically poor agreement. Moreover, our data do not suggest an association between routine use of IF or preoperative planning systems and improved IF estimation of acetabular orientation. In fact, these ICC values suggest any 2 physicians chosen randomly who perform more than 200 THAs per year would vary as much as 2 physicians chosen randomly from the survey population. Similar comparisons can be drawn for the other baseline characteristics evaluated in this survey.

We chose to evaluate the data by absolute difference so that surgeon overestimation and underestimation would not have a counteractive impact on their averaged measurement error. Although average absolute differences were within 5.9° and 8.8° for inclination and anteversion, respectively, these data showed significant variability of estimation by range and standard deviation, with some surgeons perfectly estimating acetabular inclination and/or anteversion on 1 image while estimations were off by greater than 20° on other images. This variability is blunted by analyzing the surgeons’ average estimations over the 20 survey images, but such an analysis fails to consider the potential impact on individual patients. Previous studies have reported component malposition to be a significant risk factor for early dislocation after THA, with 60% to 70% of dislocations occurring in the first 6 weeks after surgery [22,23]. Horberg et al. reported 11 (0.39%) dislocations at an average of 71 days after THA by a DAA [24]. A database review in 2018 reported dislocation readmission rates of 1.4% at a median of 40 days after the surgery for elective primary THA [25]. Although component position is not the only factor associated with dislocation, combined anteversion (acetabular plus femoral) outside of 40° to 60° has been shown to increase dislocation by an odds ratio of 6.9 [26]. Our data suggest that by IF estimation alone, 4.7% of patients have acetabular anteversion 20° or more off the true value and would thus be at significantly higher risk of dislocation. In addition to impacting dislocation rates, acetabular component positioning also affects revision rates [[27], [28], [29]]. Dislocation and revision lead to increased health-care costs and patient morbidity and stress [30]. The historical safe zones are defined as 40° ± 10° of inclination and 15° ± 10° of anteversion, and these data suggest surgeons are unreliable when utilizing only IF and should consider alternate or additional methods to optimize component position [21].

One alternative to using IF is CT-based navigation, where three-dimensional cup templates are created, thus allowing for more accurate placement and positioning of the acetabulum cup. Using this technology, Tsutsui et al. report that for both inclination and anteversion, 97.7% of the acetabular placements were within the combined target zone (30°–45° of inclination and 5°–25° of anteversion) compared to 61.3% of patients without navigation. As a result, this technology allows surgeons to achieve high accuracy of both cup alignment angles and positioning [31]. In addition to CT-based navigation, surgeons have several other options to achieve optimal component anteversion and inclination. One of which is the use of a mechanical insertion jig to assist in the alignment and positioning of the acetabular cup [28]. With advances in technology, it is now possible for patient-specific insertion jigs to be 3D printed, thus allowing for the angles of anteversion and inclination to be well within the safe zones in a cheap, effective manner [32,33]. Imageless navigation also presents surgeons with an adequate alternative to IF. Nogler et al. report that with the use of imageless navigation, there is significant reduction in the median absolute difference of inclination (1.3° to 5.8°) and anteversion (2.4° to 9.9°) when compared to component placement with visual cues alone [34]. Other studies report these navigation systems can be more difficult to place in larger patients. The impact of this difficulty appears to have a significant effect on acetabular anteversion, while inclination values remain more consistent [35]. Lastly, there is a trend toward increasing robotic assistance with many types of surgery, including orthopedics. Redmond et al. report that as surgeons increase their experience with robotic assistance for THA, procedure time decreases [36]. They also report that regardless of experience level, acetabular components are well placed with a 95% confidence interval of 8° [36]. Despite the learning curve for optimal robot-assisted THA, there is immediate and significant improvement in acetabular cup positioning when compared to IF. This contrasts the learning curve of IF, which does not demonstrate an immediate improvement, requiring the surgeon to gain experience before significant enhancement of precision is seen [29]. Additionally, Domb et al. found that 100% of robot-assisted THAs were within the safe zones for both inclination and anteversion, as described by Lewinnek et al. [21,37] All these methods provide good alternatives or additions to the use of IF to improve acetabular cup positioning with the DAA for THA. It is important to note that the use IF does minimize the variability in anteversion and inclination, but our data suggest this method remains unreliable [18].

Our study has many strengths, most notably its survey design. It is known that a poorly designed survey, without prior planning, could lead to inaccurate and misleading conclusions. Sprague et al. discussed the importance of survey design in ensuring maximum response rates from orthopedic surgeons [11]. Our survey utilized 11 of the 12 points that were presented in their article, missing only in that we did not evaluate the characteristics of nonresponders [11]. While our study has these strengths, we do recognize the limitations of this work. The participating surgeons were presented with 20 IF images and asked to analyze them, even though they had no hand in the production of those images. It is possible that each surgeon’s accuracy would increase if they were visualizing the case intraoperatively in addition to taking and viewing fluoroscopic images. This study also does not attempt to correlate surgeon accuracy with clinical outcomes and is descriptive by nature of its design. We do not correlate a surgeon’s ability to estimate component position on IF to dislocation or revision rates. Moreover, our sample size is small, and the participants are all located in a similar geographical location. We have significant risk of nonresponse bias, with a response rate of only 38%, well below the generally preferred rate of 60% [20]. However, we took measures to account for this by comparing early and late responders [20,38,39]. This analysis found that these groups were similar, thus suggesting a minimal effect of nonresponse bias [20,39]. Survey fatigue may also decrease the reliability of our results as responders may find reviewing 20 images tedious. Lastly, despite historically poor physician response rates on surveys, there is no validated evaluation tool or method to assess survey quality in orthopedics [40].

## Conclusions

Our data suggest that surgeons may not be as reliable as previously suggested with estimating acetabular cup position with IF. As technology continues to advance in the field of arthroplasty surgery, adaptation and utilization of these products may continue to improve success rates of these already highly successful procedures. We hope this survey promotes interest in improving and objectifying IF techniques to increase observer reliability.

## Conflicts of interest

Dr. A. Saxena is in the speakers' bureau of or gave paid presentations for Corin; is a paid consultant for Corin; and is a member of the Patient and Public Relations Committee of American Association of Hip and Knee Surgeons, Telecommunications Committee of Eastern Orthopaedic Association, Pennsylvania Orthopaedic Society, and Web-Based Longitudinal Assessment and Hip Program Committees of the American Academy of Orthopaedic Surgeons. Dr. E. M. Slotkin receives royalties from Corin; is in the speakers' bureau of or gave paid presentations for Corin, Naviswiss, Phillips, and RomTech; is a paid consultant for Corin, Naviswiss, Phillips, DePuy, and RomTech; is an unpaid consultant for Efferent Health; and has stock or stock options with Naviswiss, RomTech, and Efferent Health. Dr. M. Solomon receives royalties from Corin UK and Medacta; is in the speakers' bureau of or gave paid presentations for Corin; is a paid consultant for Corin; and receives other financial or material support from Corin. T. Jones is a paid employee of the Corin Group. All other authors declare no potential conflicts of interest.

For full disclosure statements refer to https://doi.org/10.1016/j.artd.2023.101109.

## Appendix A Supplementary Data

Conflict of Interest Statement for Saxena

Conflict of Interest Statement for Santana

Conflict of Interest Statement for Slotkin

Conflict of Interest Statement for Solomon

Conflict of Interest Statement for Jones

Conflict of Interest Statement for Toci

Conflict of Interest Statement for Brush
