# Supplementary material for: Surgeon Estimations of Acetabular Cup Orientation Using Intraoperative Fluoroscopic Imagining Are Unreliable
Source: Arthroplast Today. 2023 Mar 7;20:101109. doi: 10.1016/j.artd.2023.101109 (PMC10018435; doi:10.1016/j.artd.2023.101109)
Supplement: Conflict of Interest Statement for Slotkin [file mmc3.pdf]

INDIVIDUAL CONFLICT OF INTEREST STATEMENT

American Association of Hip and Knee Surgeons

(Adopted from the American Academy of Orthopaedic Surgeons disclosure statement)

The following form **must be filled out completely and submitted by each author (example, 6 authors, 6 forms). All items require a response. If there is no relevant disclosure for a given item, enter "*None.*"**

Manuscript Title

1. Royalties from a company or supplier (The following conflicts were disclosed)

Corin

2. Speakers bureau/paid presentations for a company or supplier (The following conflicts were disclosed)

Corin, Naviswiss, Phillips, RomTech

3A. Paid employee for a company or supplier (The following conflicts were disclosed)

None

3B. Paid consultant for a company or supplier (The following conflicts were disclosed)

Corin, Naviswiss, Phillips, RomTech, Depuy

3C. Unpaid consultants for a company or supplier (The following conflicts were disclosed)

Efferent Health

4. Stock or stock options in a company or supplier (The following conflicts were disclosed)

Naviswiss, RomTech, Efferent Health

5. Research support from a company or supplier as a Principal Investigator (The following conflicts were disclosed)

None

6. Other financial or material support from a company or supplier (The following conflicts were disclosed)

None

7. Royalties, financial or material support from publishers (The following conflicts were disclosed)

None

8. Medical/Orthopaedic publications editorial/governing board (The following conflicts were disclosed)

None

9. Board member/committee appointments for a society (The following conflicts were disclosed)

None

Each author must sign AND print or type his/her name, date and submit a separate form

In addition, one BLINDED Conflict of Interest form (no author names used) should be submitted per manuscript with all author disclosures.

Eric M. Slotkin, DO	[signature]	8/25/2022
Author Name (Print or Type)	Author Signature	Date
